# A review and framework for understanding the potential impact of poor solid waste management on health in developing countries

**DOI:** 10.1186/s13690-016-0166-4

**Published:** 2016-12-26

**Authors:** Abdhalah K. Ziraba, Tilahun Nigatu Haregu, Blessing Mberu

**Affiliations:** African Population and Health Research Center, P. O. Box 10787-00100, Nairobi, Kenya

**Keywords:** Solid waste, Municipal waste, Health impact, Urbanization, Hazardous waste, Conceptual framework

## Abstract

**Background:**

The increase in solid waste generated per capita in Africa has not been accompanied by a commensurate growth in the capacity and funding to manage it. It is reported that less than 30% of urban waste in developing countries is collected and disposed appropriately. The implications of poorly managed waste on health are numerous and depend on the nature of the waste, individuals exposed, duration of exposure and availability of interventions for those exposed.

**Objective:**

To present a framework for understanding the linkages between poor solid waste management, exposure and associated adverse health outcomes. The framework will aid understanding of the relationships, interlinkages and identification of the potential points for intervention.

**Methods:**

Development of the framework was informed by a review of literature on solid waste management policies, practices and its impact on health in developing countries. A configurative synthesis of literature was applied to develop the framework. Several iterations of the framework were reviewed by experts in the field. Each linkage and outcomes are described in detail as outputs of this study.

**Result:**

The resulting framework identifies groups of people at a heightened risk of exposure and the potential health consequences. Using the iceberg metaphor, the framework illustrates the pathways and potential burden of ill-health related to solid waste that is hidden but rapidly unfolding with our inaction. The existing evidence on the linkage between poor solid waste management and adverse health outcomes calls to action by all stakeholders in understanding, prioritizing, and addressing the issue of solid waste in our midst to ensure that our environment and health are preserved.

**Conclusion:**

A resulting framework developed in this study presents a clearer picture of the linkages between poor solid waste management and could guide research, policy and action.

## Background

Solid waste management is a growing challenge to many rapidly urbanizing areas in Africa. It is currently estimated that the rate of urban solid waste growth is faster than that of urbanization. Global estimates indicated that by 2002, 2.9 billion urban residents generated about 0.64 kg of waste per person per day and by 2012, this rose to 1.2 kg per person per day with a total urban population of 3 billion. Currently, it is projected that by 2025 there will be about 4.3 billion urban residents who on average will generate 1.42 kg of waste per day [1]. It is known that solid waste has effects on health and it is one of the major reasons why solid waste management is a top environmental and public health issue. However, while several causal linkages between exposure to waste and health outcomes for particular types of waste are well established, others remain unclear or not prioritized as public health issues. In cases where the causal linkages are known, the full extent of the burden of ill health attributable to exposure might not be known. Part of the challenge in establishing the causal linkages is the difficulty in unambiguously ascertaining the type, the dose and duration of exposure [2]. On the side of health outcomes, the challenge is the difficulty in ruling out other causes since other exposures in the environment might potentially cause the same outcomes [3, 4]. Additionally, some clinical outcomes such as cancers and other forms of degenerative disorders take long to manifest after exposure and loss to follow up of exposed individuals is a common challenge [5–8].

### Urbanization and solid waste generation

Solid waste generation and urbanization are intimately related and therefore it is important to briefly reflect on the urbanization phenomenon in the region. In 1950, about 30% of the world’s population lived in urban areas. It is currently estimated that by 2050, about 66% of the world’s population will be living in urban areas. Sub-Saharan Africa is urbanizing at a faster rate than any other part of the world. While Africa is still the least urbanized (40%), it is estimated that by 2050, about 56% of the population in Africa will be living in urban areas [9]. Going by the current trends, urbanization is a phenomenon that is rapidly growing and urban centers will remain the engines for economic growth and associated waste generation. Urban centers will also bear a substantial burden of ill-health in the coming decades attributable to poor waste management. While the per capita waste generation is highest in the developed world, these countries have better waste management practices that mitigate against potential adverse health impacts. In countries that are rapidly urbanizing and developing economically such as China and India, the ever increasing volumes of waste generated and weaker waste management practices poses serious health risks [1]. Human activities and their products are now recognized as the main cause of current global environmental and climatic changes that have direct effects on health and wellbeing [10]. Similarly, at a local municipal level, many human activities generate waste and these are major causes of environmental and health challenges including infectious diseases such malaria, cholera, dysentery, respiratory complications and injuries among others [11–16]. The growing urban population means more solid waste, and higher impact on environment and health. Increased solid waste results into increased demand on existing solid waste management services, which are in many African countries, the single largest budgetary item for local governments [1].

The urban growth in most of Africa has not been in synchrony with expansion of social amenities and economic opportunities, with many cities struggling to provide basic services such as shelter, water and maintaining a clean environment amidst an ever growing but largely poor urban population [17–19]. Urban centers have been considered places of opportunity, wealth, better education and health. Indeed, from the health perspective, urban populations have historically had overall better health indicators compared to rural populations and this became to be known as the urban health advantage. In the face of new urban challenges, the urban health advantage is waning [20, 21].

The overarching objective of this paper is to develop and present a framework that aids understanding of (poor) solid waste management and its impact on health with a view to stimulate research, guide development of policies and implementation of appropriate interventions. More specifically, this study identifies and describes the main pathways through which poor SWM affects health; and sheds light on how the pathways can be exploited by different actors to reduce potential impact on health and wellbeing. The development of the framework was informed by a review of the literature contextualized in a developing country (level of socio-economic development and urbanization, health realities, and national and international responses to the challenges).

## Methods

The review of the literature on the impact of poor solid waste management on health was the first major step in identifying, synthesizing, and integrating relevant evidence. The evidence was then used to create and critique a framework that summarizes and shows interlinkages and possible pathways through which exposure to solid waste may be detrimental to health. Both peer-reviewed and grey literature was searched. For the peer-reviewed and indexed literature, Pubmed online library was searched using a pre-determined search strategy. The following search word combinations: Solid/municipal waste; Solid/municipal waste management (generation, disposal); health impact/effects were used. Retrieved articles were reviewed for relevance. Similar search terms were used to search grey literature from Google Scholar. A configurative synthesis of the evidence was conducted to develop the framework and took it through a series of consultative reviews and expert informed revisions. The review summarized below starts with defining solid waste including classification, solid waste management practices, challenges and opportunities and lastly identifying the linkages/associations between solid waste and adverse health outcomes.

## Literature review

### Defining solid waste

Solid waste may be defined as all discarded solid materials resulting from households, industrial, healthcare, constructional, agricultural, commercial, and institutional sources. Solid waste generated in a city is often referred to as municipal solid waste. In other literature and jurisdictions this category may exclude sewage, dissolved solids in water, and industrial waste [1]. For this paper, no exclusions were made for the reason that in most developing countries, most of the solid waste is not sorted at source, collection, transportation and disposal points [22]. Thus, municipal waste in the context of developing countries may include waste that would not ordinarily be considered municipal waste. Solid or municipal solid waste management refers to the planning, financing and implementation of programs for solid waste collection, transportation, treatment and final disposal in an environmentally and socially acceptable manner [23]. Failure to adhere to set standards at any of the various stages constitutes “poor solid waste management”.

### Solid waste management practices

Solid waste management practices greatly vary across regions, countries and even within country [1, 24]. Modern waste management approaches encourage reduced waste generation, re-use, recycling, composting, and safe disposal through landfills, however, these are often not practiced. In developing countries a large proportion of waste is not re-used. Waste sorting is also rare and therefore this makes it difficult to re-cycle or compost. As a result a large proportion of solid waste in developing countries is disposed of on open dump sites and many times burnt [1, 2, 24–26]. The variations in waste management practices are often a reflection of existence of laws and policies governing waste management and extent of their enforcement, available funding, composition and quantity of waste generated [1]. In many developing countries, solid waste management is the responsibility of both the municipal authorities and private providers [1, 24, 25, 27]. Collection is often from source or temporary dumping ground, and final disposal is often at an open dumping site on the outskirts of the city. Dumping sites are often sprawling open grounds where truckloads deposit the waste. Dumped waste is often scavenged for usable articles, recyclable materials and many times burnt to reduce the bulk. Due to limited solid waste sorting at any stage, solid waste composition is complex and may contain industrial, medical, electronic, and human waste dumped on the same open grounds where all the other municipal waste is dumped [28, 29].

### Waste classification

Municipal solid waste is often categorized into two major groups: organic and inorganic. The organic municipal solid waste can further be divided into three categories: putrescible, fermentable, and non-fermentable. Putrescible wastes include products such as foodstuff that decompose fast. Fermentable wastes decompose rapidly, but without the unpleasant accompaniments of putrefaction while non-fermentable wastes tend to resist decomposition and, therefore, break down very slowly. Inorganic solid waste includes articles like metals, plastics, and other non-biodegradable materials. In terms of toxicity, some solid wastes are classified as hazardous including pesticides, medical waste, electrical waste, herbicides, fertilizers and paints and are recommended to be disposed of in special ways and not to be mixed with general municipal waste [24]. Solid waste in developing countries characteristically has a high content of organic matter compared to that in developed countries. For example, studies conducted in the region estimated that in Juba South Sudan, organic waste constituted about 31% of all waste by weight [30], 61% in Ghana [31] and 54% by weight in an Ethiopian town Jimma [32]. The high organic content has implications for waste management including recycling, but also a potential source of ill-health if mismanaged.

### Solid waste and the wider development agenda

For health, environmental, and economic reasons, management of solid waste is and should be an important undertaking in any urban setting. There are wide variations in policies and practices in solid waste management between regions, countries, large and smaller cities and formal and informal areas within a city. While all urban centers face similar solid waste management challenges, the impact vary depending on how policies and practices are implemented. From the global development agenda perspective under the auspices of the Millennium Development Goals (MDGs), ensuring environmental sustainability (MDG 7), was identified as a key area. Review of progress on this MDG shows that an estimated 2.1 billion people gained access to improved sanitation between 1990 and 2015; elimination of ozone depleting substances; proportion of global population using open defecation halved since 1990; and proportion of urban population leaving in slums fell from 39.4 to 29.7% between 1990 and 2014 [33].

Going into the new global dispensation, the Sustainable Development Goals (SDGs), the relevance of the issue of protecting the environment and preserving health through proper solid waste management in cities has become even more pronounced. The SDG agenda advocates for reduced generation of waste, and increased reuse and recycling. It touches on SDG3 *(health lives and promote well-being)*; SDG6 *(water and sanitation)*; SDG11 *(making cities inclusive, safe, resilient & sustainable)* and SDG 13 (combating climate change and its impact) [34]. SDG 11, specifically has an indicator that relates to solid waste management: “*percentage of solid waste regularly collected and well managed*”. However, like other prior social development agendas, the challenge may be located in the operationalization and implementation. In many countries in the developing world, management of solid waste is not mainstreamed, poorly funded and has always fallen below expectation [1, 35, 36]. A review of evolution of policies, show that, Kenya for example, has made numerous efforts supported by policies, to manage solid waste in a sustainable way but in most cases implementation has been haphazard and fallen short [37]. The potential consequences of this failure to manage solid waste forms the heart of this paper as illustrated in the framework, with particular focus on the health impacts.

### The conceptual framework

The interlinkage between poor solid waste management and adverse health outcomes may be overt and direct but may also be indirect and not obviously linkable to poor health outcomes of a population. This paper presents a framework to aid understanding the interlinkages between poor solid waste management and health, and gives the rationale for maintaining proper solid waste management as an investment in preventing ill-health and promoting wellbeing. The paper discusses various concepts related to solid waste management, and how these independently and jointly impact on urban health making reference to developing country contexts. The concepts discussed are not entirely new but are applied to a context of a developing country urban setting where the challenge of solid waste management is growing without commensurate interventions to manage it. Finally, a discussion and interrogation of the interlinkages and pathways between solid waste and the ill-health and how these can be exploited for implementation of cost effective interventions is provided.

The literature supporting the framework is summarized in two major categories: exposure to solid waste and the mechanism that bring about adverse health outcomes; and adverse health impacts. Under exposure, five categories of how individuals can be exposed are considered including: i) exposure to waste by waste generators; ii) exposure from handling waste among waste collectors; iii) pickers at dump sites; iv) living/working in neighborhoods of dumping sites and incinerators; and v) accumulation noxious substances such as heavy metals in the environment and subsequently in the food chain.

## Exposure to solid waste

Exposure to solid waste may be obvious but may also be occult. Exposure to solid waste may take the form of bodily contact, penetrating injuries, inhalation, or ingestion. Exposure to solid waste is a function of how much solid waste is generated, how it is collected, transported, and the proportion disposed of safely [1, 25, 38]. It is estimated that in developing countries, waste generated per capita per day is about 0.65 kg compared to 2.2 kg in Organization for Economic Cooperation and Development (OECD) countries. The African region contributes about 5% of solid waste generated globally, 44% by OECD and 12% by Latin America and the Caribbean [1]. Solid waste collection in low income countries is less than 50% compared to about 98% in high income countries and in most cases disposal is at open dumpsites or land fill with limited organized recycling [1]. At a higher level, risk of exposure to solid waste is influenced by presence or absence of good policies and allocation of financial resources to manage it. Categories of people exposed to solid waste range from those who generate the waste, those who collect waste it, such as the municipal workers, those who pick waste for a living and those living or working near disposal site such as landfills or dump sites and incinerators. The literature reviewed here assesses the exposure to solid waste, the knowledge of exposure, risk perception and mitigation practices among the various actors outlined above.

### Exposure to solid waste among waste generators

Exposure to solid waste may occur right from the point where the waste is generated [39]. A good example is the medical waste. Medical personnel and hospital housekeeping staff are at higher risk of exposure to waste and infection from biological waste [39, 40]. While medical waste requires stringent management, it is not uncommon to find medical waste being handled like household waste [36]. Sharp used medical equipment such as needles and scalpels are supposed to be disposed of in a safe “sharps “container but this is not always followed. Needle stick injuries from misplaced used needles are a common occurrence among health care providers [39]. Additionally, other than penetrating injuries or cuts, medical waste and contaminated surfaces may have contain highly infectious microbial agents such as ebola virus and hepatitis B & C virus which can be transmitted to exposed workers [41, 42]. Other forms of exposure to waste by generators may include industrial workers who do not wear protective gear and are at risk of getting exposed to waste generated from their workplace such as toxic chemical waste.

### Exposure to solid waste among collectors

Occupational exposure to solid waste is a constant risk waste handlers are faced with. Exposure can happen depending on the level of protective ware, knowledge of risk, standards and practices of waste sorting and equipment available to such workers [15]. In many of the developing countries, municipal waste (which is a mixed bag of waste) is handled by cheaply hired workers with limited protective gear and limited appreciation of the risk involved in handling solid waste [15]. Often they also have no legal protection and recourse in case of injury as their engagement terms are largely non-binding. Even where there are binding working relationships between the waste handler and employer such as the municipal councils, the challenge is that some of the effects of exposure may manifest long after the working relationship ceased to exist. The near absence of waste sorting and lack of protective wear put waste handlers at very high risk of exposure [36, 43]. This is particularly important in developing countries where solid waste is often mixed with high risk waste such as medical waste especially from small facilities being disposed of as general municipal waste [36].

### Living in neighborhoods of dumping and incinerator sites

In addition to enduring the nauseating and pungent smell and the unpleasant sight of rampant scavenging animals at dump sites, residents in the neighborhood of dumping sites have an ever-present risk of infection transmission through vectors and rodents that are abound at dump sites and inhalation of fumes from the burning waste [44, 45]. The decomposing and festering solid waste attracts all manner of vectors including common houseflies that are very efficient in transmitting disease causing germs. Children living in such neighborhoods are exposed to a triple risk infectious diseases, injury and inhalation of dangerous fumes from the continuous burning of waste. However, due to the difficulties involved in quantifying the “dose” of exposure, the evidence linking residence near landfills and or dump sites and health outcomes remains weak [4, 46].

Many poor urban residents do not get their water supply from the main municipal sources. Water from shallow unprotected wells are often contaminated by leachate from dumpsites. Still even those who draw water from the municipal sources may get it from illegal connections that are susceptible to breakage and contamination. Other common sources of water include protected or unprotected springs. In such circumstances, potentially the risk of water contamination from waste disposed of upstream is high. Improper human fecal matter and waste from abattoirs disposal is poor in many places and yet these are a rich source of disease-causing bacteria posing a serious health risk to individuals using such contaminated water [47, 48]. On the other hand it is often the case that solid waste containing noxious chemicals at dump sites is burnt and this process may produce toxic fumes which cause respiratory complications and allergic reactions in some people.

Incineration is recommended for disposal of certain types of waste. However due to lack of appropriate equipment and fuel, incinerators are often not well run, for example not maintaining the right temperature, might result into releasing of noxious fumes in the environment [49–51]. It has been reported that living in the neighborhood of incinerators that are not well run and protected poses high respiratory disease risk [6]. Those operating the incinerators are also at risk of these health challenges as occupation hazards [8].

### Exposure of solid waste to pickers and recyclers

In many African cities, solid waste dump sites are located on the outskirts of the city which are also home to a huge urban poor population often living in slums with no proper means of livelihood. Dumping sites are a source of economic livelihood to many who pick and retrieve articles that they consider valuable to them or the market for direct use or recycling [25, 52]. Retrieved articles range from clothes, household utensils, food, ornaments and scrap metal and plastics among others. The process of picking waste exposes such people to many risks including infection, respiratory complications from fumes and injury from sharp objects [25]. Retrieved articles and food that find their way to the market puts a huge population at risk. In settings where women are the majority in the informal sectors, they are likely to also be over represented in the waste picking business. Similarly, get involved in waste picking and are likely to get disproportionately affected by injuries, respiratory complications and infections. Solid waste recycling has also been associated with health risk including physical injury, infections, and inhalation of particulate matter including bioaerosols [29, 53].

### Accumulation of noxious chemicals in environment including food chain and air

In most of the developing world, sorting of waste is hardly practiced. Waste that by law is supposed to be managed in a stringent manner finds its way on dumping sites for general waste. In a study involving assessment of management of medical waste in 5 hospitals, it was reported that there was no sorting of waste and yet 26.5% of the waste was categorized as hazard [36]. Industrial effluents often discharge into rivers while medical waste is often mixed with household waste as well as electronic waste. Petroleum products including paints laden with lead are discharged in open spaces or water channels. While some of the chemicals discharged might have short-term effects on animal and plant life, others are carried through the food chain where they accumulate and have deleterious effects much later. Heavy metals such as lead, arsenic and mercury are of particularly high public health importance yet no clear measures are enforced to control their disposal and help limit environmental contamination [54–56]. Poorly managed solid waste disposal systems such as in compositing, sewage treatment and poor constructed landfills can all lead to environmental contamination and consequent exposure to the general public [2].

## Health impacts of exposure to solid waste

The impact of solid waste on health is varied and may depend on numerous factors including the nature of the waste, duration of exposure, the population exposed, and availability of prevention and mitigation interventions [13, 15, 44]. The impacts may range from mild psychological effects to severe morbidity, disability or death. The literature on health impacts of solid waste exposure remains weak and inconclusive in many cases due the difficulties encountered in accurately ascertaining exposure, controlling for confounders, accounting for duration of exposure and inability to follow up those exposed to ascertain outcomes that do not manifest in the short term [5, 57]. This notwithstanding, the literature review presented here sheds light on several pieces of evidence linking solid waste exposure and self-reported outcomes but also those where ascertainment of exposure and health outcomes were empirically confirmed.

While certain health impacts might be immediate, obvious to discern and directly linkable to the solid waste exposure, others may be occult, longer term and difficult to attribute the effects to a particular type of waste [4, 45, 58]. This makes establishing the burden of disease attributable to solid waste and full epidemiologic spectrum of diseases emanating from the exposure a difficult undertaking often requiring large sample sizes and prolonged periods of follow-up [7, 46, 57]. Surveillance data are lacking due to the complexity involved in measuring exposure and outcomes but also the limeted programmatic focus and funding to this area. While estimating the exposure and the outcomes are difficult, available research allows us to conceptualize and draw linkages on how current solid waste exposures might be contributing to the observed ill-health at individual and population level. This may not only guide designing of more elaborate studies, but also guide policies and interventions.

**Fig. 1:**
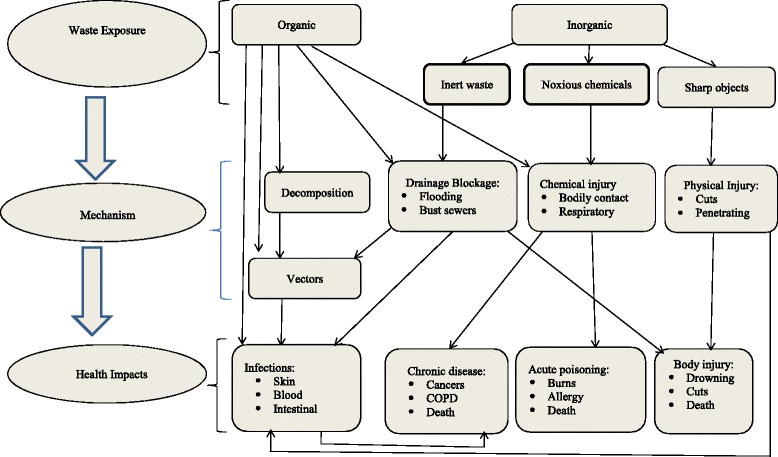
A framework for understanding the linkages between poor solid waste management and adverse health outcomes

Figure 1, is a schematic conceptual representation of the linkages between exposure to the various types of solid waste, the pathways to negative outcomes and final impact on health. The representation here is only illustrative and not exhaustive. For ease of understanding, health impacts have been categorized into four:

- Infection transmission: This could be bacterial, viral and other disease causing organisms
- Physical bodily injury: These may include cuts, drowning, blunt trauma, and chemical or radiation injury. This may range from immediate skin or inhalation burns, to longer terms effects.
- Non-communicable diseases- long term exposure may lead to cellular damage and development of cancer while other might result in bodily organ injury and damage.
- Emotional/psychological effects (strong smells, unsightly waste as human body parts)

One type of solid waste may lead to more than one health outcome directly or through an intermediate mechanism for example through vectors and other individual level predisposing factors.

### Infections

Poorly managed medical waste, is a major source of infection for patients, health care workers, waste handlers and general public [14, 59, 60]. Where all medical waste is properly disposed of, the risk of infection to the general public is limited, but remains substantial to providers and their clients. While protocols on handling medical waste exist in many settings, their implementation varies from one place to another depending on how stringently prevention of infection protocols are implemented and observed. Indeed many health care personnel and medical waste handlers do not use personal protective gear [14, 35, 61, 62].

A variety of pathogenic organisms are transmitted from biological specimen, contaminated medical waste and sharp medical objects such as hypodermic needles. Hepatitis B infection is a common infection often transmitted through skin cuts, mucous membranes, needles stick injures and contaminated surfaces [39, 41, 43]. Although it is recommended that all used and disposable sharp equipment should be discarded in a sharps containers, these are often not available resulting into many health personnel getting needle stick injuries. The risk of transmission of infection from medical waste is substantial including hepatitis B, ebola and Hepatitis C among others [40, 59]. Other important pathogens that can be transmitted from medical waste include pathogenic bacteria such one that causes tuberculosis, anthrax, pneumonia, meningitis, and infections of the gastro-intestinal system. Evidence shows that workers who handle medical waste are at a higher risk of nosocomial infections [14, 43].

Decomposing organic waste is a rich medium or culture for growth of numerous micro-organisms many of which are diseases causing if passed on to humans. Also there is always a risk of transmission through vectors such as houseflies but also through human contacts as is the case with waste handlers who do not use protective wear and waste pickers who most of the time use bare hands [12, 13, 63]. Additionally, articles retrieved from waste may be sold to unsuspecting public without undergoing thorough cleaning hence posing a risk of infection transmission.

Gastro-intestinal infections such as typhoid fever, polio virus infection, hepatitis E infection, and cholera are often transmitted through contaminated food or water [11, 13, 38]. Toilet ownership in Kenya, for example, is very low with 12% of all households not having any form of toilet [64]. Even those households with a toilet, many are not connected to the main sewer line. These result into fecal matter being disposed of in open spaces while other households do not have any form of toilet and thus dispose of fecal matter as general waste, popularly referred to as flying toilets or discharged into rivers [65]. Human fecal matter is a known source for pathogenic enteric parasites, typhoid fever infection, polio virus infection, hepatitis E infection, cholera and common gastroenteritis transmitted human contact, vectors or contaminated water [11]. Studies have revealed high levels of pathogenic parasites in dump site waste confirming the risk waste handlers and pickers are exposed to [63]. This challenge of proper feacal matter management is not limited to households but also institutions such as hospitals and schools. There are reports of cholera outbreaks emanating from fecal waste coming from a hospital [66, 67].

### Injury

In many developing countries, the practice of sorting waste at source is almost non-existent even for high risk waste such as sharps generated from medical facilities [32, 68]. Presence of sharp objects in waste poses a high risk of injury to both those who generate the waste, the handlers and pickers [15, 16, 69]. Poorly disposed surgical blades, needles frequently injure medical workers, medical housekeepers and waste collectors of medical waste while sharp objects such broken glass injure domestic workers and waste handlers. Where waste is disposed of in open dump site accessible to pickers, the risk of injury from sharp objects is ever present [16].

Urban floods are common in many cities. While poor urban physical planning may be largely to blame for the increasing phenomenon of urban floods, partly the problem can be attributed to rampant blockage of drainage systems by solid waste [70–72]. Inappropriate disposal of waste, especially the non-biodegradable plastic paper bags results into these being swept downstream resulting into blockage of drainage systems. Floods not only destroy property, they have claimed lives both on roads and homes and damage sewerage systems leading to wide spread environmental contamination with human waste and associated risk of infection transmission [66, 73]. Blocked drainage systems are also breeding sites for diseases transmitting vectors such as mosquitoes.

Injuries from chemicals can be in the forms of skin burns, inhalation burns, explosions and intoxication. Fumes from burning chemicals at dump sites or from incinerators may cause respiratory, allergic and other complications [3, 15]. Pharmaceutical and industrial chemicals are often not disposed of appropriately and at times get back into the market. Obsolete pesticides, old batteries, among others contain chemicals that are dangerous to human life yet are often disposed of just like common litter [74]. Medical waste may also include substances that are cytotoxic and or carcinogenic. Improperly disposed substances and equipment may result is disastrous effects to the public as was the case with the caesium-137 irradiation accident from a disused radiotherapy unit in Goiania, Brazil [75].

### Chronic diseases (From long term exposure to chemicals and infections)

While many international protocols and guidelines on disposal of hazardous chemicals exist, these are not strictly adhered to especially in this part of the world. Environmental contamination with chemicals from industries is common endangering both humans and wild life. It is a common practice for industries to discharge their waste into rivers. It is also a common practice to dispose e-waste on open dump site. These are often burnt to retrieve desired components and yet the fumes are hazardous [28, 29]. The world is dependent on petroleum for energy and industrial applications. Disposal of petroleum waste is poor and yet has long lasting impact on humans and the eco-systems in general [76]. Medical and pharmaceutical chemical waste, often include antibiotics, vaccines, and radioactive substances. In Brazil, a disused and vandalized for scrap radiotherapy unit caused accidental and prolonged exposure to radiation from caesium-137 leaving many with severe health problems while others died [75]. Common industrial waste contain dangerous chemicals such as lead, arsenic and mercury among others [77]. These chemicals may affect health through direct contact while others are through accumulation in the food chain [54]. Genotoxic substances cause changes in the internal cell structure. This change may or may not result in cancerous change. However, is strong evidence linking exposure to noxious substances and development of different types of cancer including lung cancer, bladder cancer, skin cancer and reproductive tract cancers among others [5, 78, 79]. Similarly, radioactive substances and their radiation are known to cause cell damage and may result into different forms of cancer. Prolonged exposure and inhalation of noxious, irritant or volatile chemicals, may lead to the respiratory system becoming hyper-sensitized and this may result into chronic obstructive pulmonary disease [80, 81].

### Psychological/Emotional impacts

Residents living next to dumpsites are usually affected by stench, the sight of marauding scavenging animals and social stigma. In extreme cases, solid waste has been reported to contain human body parts or aborted fetuses which may be distressing and could affect the mental well-being of the residents and those involved in waste picking. Moreover, for those who live closer to the dumping sites, the nuisance of scavenging animals and birds ﻿may affect their emotional and psychological health [4, 44]. Heavy metal poisoning has also be associated with mental disorders [58, 82].

## Discussion

As developing countries continue to grow economically, so does urbanization and the challenge of solid waste management. Municipal solid waste is a recognized environmental and health challenge but also an economic resource on which thousands of individuals eke a living through picking, re-using and recycling. While the per capita generation of solid waste per day is lower in developing countries, this is rapidly increasing and this is happening when there is limited expansion in the capacity, innovation, and funding to handle the challenge [1]. Characteristically, in most countries in Africa, solid waste has a high organic content making it a fertile medium for pathogens to thrive [1, 31, 32]. Secondly, solid waste is rarely sorted making recycling difficult but also more hazardous to handle from point it is generated to final disposal. Furthermore, in general, less than half of all solid waste in low income countries is collected implying that a large fraction of waste is disposed of in unsafe ways posing health risks to the general public. Lastly, even the collected waste is inadequately handled. Open dumpsites are a common disposal method and this poses serious environmental contamination risks, but also act as sources of diseases vectors and pathogenic agents [44, 47, 56]. Due to poor handling and maintenance, even disposal methods that are deemed safe in other setting can be hazardous in poor countries. Poorly maintained and run incinerators pose a health risk not only to the operators but also to those living in the neighborhood due incomplete combustion and subsequent release of dioxins [50].

Given the high risks of exposure to solid waste, it expected that there is a corresponding high burden of adverse health effects and mortality attributable to solid waste. The framework clarifies many of the linkages, but definitely not exhaustive due to lack of knowledge of causal linkages while in other cases where the linkages are known, the burden of the impact is not clear to many including policy makers. However, in spite of these challenges, existing evidence on the need to appreciate the health risks associated with various types of solid waste is strong and can be a good basis for drawing more attention on improving solid waste management. There is compelling evidence to show that solid waste, especially medical waste and other biodegradable waste are potential sources of pathogenic organisms such as viruses, bacteria and fungi and as such need to be strictly managed. This has been demonstrated among handlers of medical waste, pickers of solid waste and those living in the neighborhood of dumping sites. Prevalence of Hepatitis B and C virus infection is much higher in groups exposed to waste compared to the general population [14, 39, 43].

Toxic substances such as heavy metals and other noxious gases that are known to cause degenerative changes in tissues are often found in higher concentrations from disposal sites or incinerators fumes [5, 6, 81]. These types of waste are increasing with socio-economic development and industrialization. While definitive causal linkages between these exposures and cancers, chronic obstructive pulmonary disease and other generative disorders may still remain elusive, there is enough reason to take action to reduce risk of exposure to solid waste [4, 7, 8]. In addition to the poor solid waste collection and disposal practices, mitigation against known risks are also limited [14]. Handlers of medical waste can benefit from consistent use of personal protective equipment, and vaccination against the certain infection such hepatitis B virus. These can be ensured through legislation enforcement, health education to all those involved in the solid waste management chain, and provision of vaccinations to those at risk and provision of treatment to those already affected.

## Conclusions and recommendations

From the literature is clear that there is a strong linkage between poor solid waste management and adverse health outcomes. A broad spectrum of groups of individuals are at risk of ill-health emanating from poor solid waste management. As the volume of waste generated increases with urbanization and industrialization, so does the complexity and content of the waste. The effects of some of the noxious waste will only manifest several years after exposure. The existing policies are not encompassing enough and their implementation is far from addressing the challenge [27, 83]. Interventions aimed at protecting workers including use of protective wear and the public are not fully implemented and this leaves many at-high-risk populations not protected [84, 85].

Due to weak implementation, existing policies and interventions, surveillance is almost non-existent. Existing research is also limited particularly in assessing exposure risk and health outcomes. Recognizing the extent of the challenge, and acknowledging the limited resources, there is need to engage strategically at various levels to generate evidence that will help highlight the problem and also feed into advocacy plans for sensitization of the public, public health officials, employers and all those at heightened risk of ill-health from solid waste. This framework can be used as an advocacy tool to demand for sensitization at all levels including policy, researchers, employers, waste handlers and the general public. This is important because the effects are not limited to those handling, picking or living near disposal sites. It is a health challenge for the general public and requires a well-grounded approach to ensure that all waste is managed and disposed of in a safe manner. Based on the foregoing discussion, the following recommendations are proposed:

1. Waste management should be prioritized as a social service, with adequate budget lines. Important to note that allocating money to waste management will not translate into better results unless there is adequate sensitization, good fiduciary practices and accountability.
2. Engage several stakeholders in the management of waste to generate a sense of responsibility and interest from all stakeholders.
3. Individuals involved in waste management should always wear recommended protective gear. This is partly the responsibility of employers but employees also need to be sensitized on the need to adhere to safety precautions.
4. Public education on individual citizen’s role in ensuring that waste is appropriately managed. Simple actions such not littering on the road, can go a long way in ensuring a cleaner environment. Gradual introduction of more concrete actions such as waste sorting at point of generation will go a long way in improving solid waste management.
5. Moving from policy to comprehensive implementation plan drawing on success stories from other countries. A starting point is to characterize waste, adapt good waste management practices, and promote use of technology in activities such as energy generation from waste.
6. Waste is not useless! The culture of recycling should be encouraged. Recycling can help in reducing volume of waste, and reduce need for exploitation of raw materials. For example, the growing demand of plastics means more petroleum is needed which comes with a cost but also impact on the environment.

## Abbreviations

COPD: Chronic obstructive disease
MDG: Millennium Development Goals
OECD: Organization for Economic Cooperation and Development
SDG: Sustainable Development Goals
UNEP: United Nations Environmental Program
